# Development of systemic and mucosal immune responses against gut microbiota in early life and implications for the onset of allergies

**DOI:** 10.3389/falgy.2024.1439303

**Published:** 2024-07-17

**Authors:** Anna-Lena Pirker, Thomas Vogl

**Affiliations:** Center for Cancer Research, Medical University of Vienna, Vienna, Austria

**Keywords:** microbiome, early life, allergy, antibodies, pregnancy, breast milk

## Abstract

The early microbial colonization of human mucosal surfaces is essential for the development of the host immune system. Already during pregnancy, the unborn child is prepared for the postnatal influx of commensals and pathogens via maternal antibodies, and after birth this protection is continued with antibodies in breast milk. During this critical window of time, which extends from pregnancy to the first year of life, each encounter with a microorganism can influence children's immune response and can have a lifelong impact on their life. For example, there are numerous links between the development of allergies and an altered gut microbiome. However, the exact mechanisms behind microbial influences, also extending to how viruses influence host-microbe interactions, are incompletely understood. In this review, we address the impact of infants’ first microbial encounters, how the immune system develops to interact with gut microbiota, and summarize how an altered immune response could be implied in allergies.

## 1 Introduction

The gut microbiome is composed of a diverse community comprising bacteria, archaea, eukaryotes, and viruses. These microorganisms maintain intricate relationships, not only among themselves but also with the human host, spanning from symbiotic to parasitic interactions, thereby exerting a profound influence on the host's immune system (1). This crucial interplay between the human immune system and these microbial communities begins early in life, extending influences into adulthood (2).

**Figure 1:**
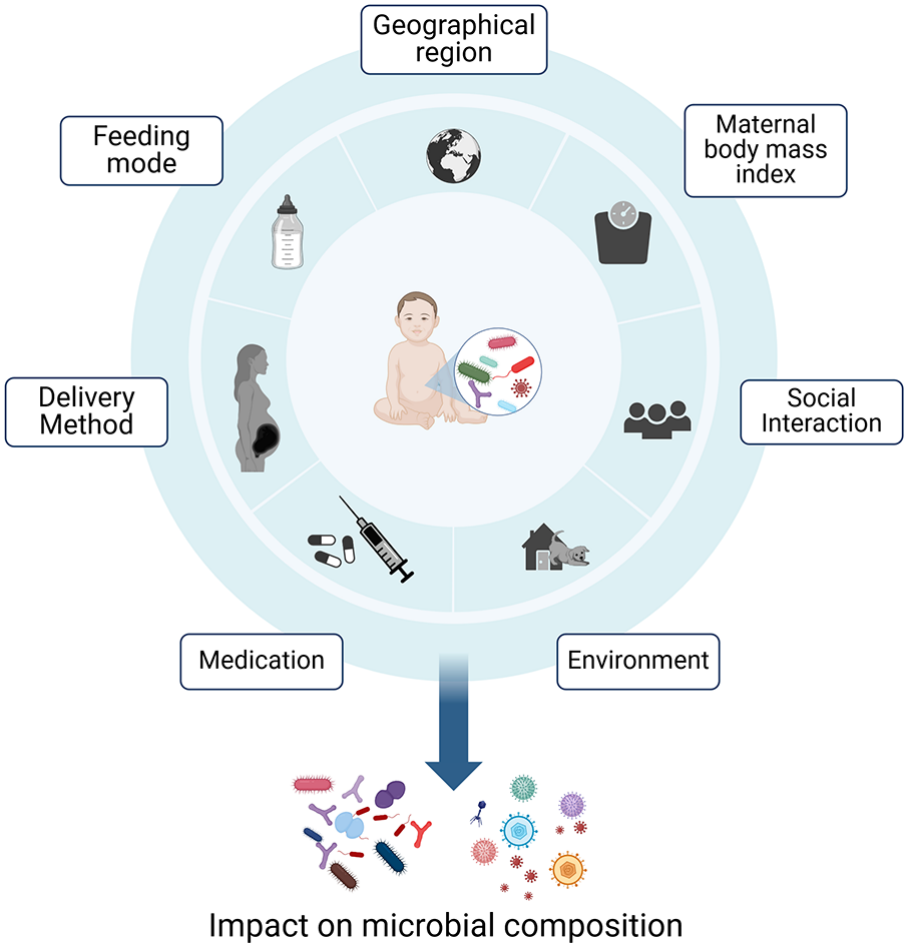
Early life factors influencing the composition of the gut microbiome. The microbial composition is influenced by feeding mode, medications, maternal-, geographical- and social factors. Host-microorganism interactions in prenatal and early postnatal life can have permanent effects on the immune development (5–7, 9, 10). Created with BioRender.com.

The preparation of the immature immune system for exposure to this vast number of microorganisms already begins *in utero*. Here, IgG antibodies, which are the only antibody class able to be transferred via the neonatal Fc receptor (FcRn), cross the placenta to the umbilical cord, reach systemic circulation in the infant (3). After birth, primarily IgA antibodies are transferred from the mother to the infant via breastmilk. These antibodies support the infant in keeping the balance between protection from pathogens by providing passive immunity and tolerance to non-threating, beneficial commensals (4). The human gut microbiota in early life is shaped by various factors, including the mode of delivery, feeding mode (breast milk vs. formula), antibiotics treatments, maternal health status prior to conception, maternal body mass index and diet, as well as the geographical region (Figure 1) (5–10). During the first years of life, the composition of the microbiome exhibits the highest levels of variability and host-microorganism interactions in prenatal and early postnatal life can have permanent effects on the immune development. The microbiome composition ultimately stabilizes into an adult-like configuration around the age of three (9, 11).

The most critical time period therein, is the switch from being breastfed to the introduction to solid food at approximately six months of age (12). This developmental period, known as the “window of opportunity”, in which the microbial diversity is settling, could be seen as a double-edged sword (13, 14). While it creates a fertile ground for microbial colonization, it also leaves infants more vulnerable to external factors that can disrupt the delicate balance of their microbiota. These external factors disturbing the microbiota include antibiotics and malnutrition, which can have long-lasting adverse effects on the function of the immune system (15, 16). Any dysbiosis could therefore result in the development of diseases including necrotizing enterocolitis, inflammatory bowel disease, obesity and allergy (17–21). Interestingly, also allergic diseases have been associated with microbial dysbiosis and over the last years, several studies have suggested an involvement of gut microbiota in the development of allergies and asthma (22–24). These effects have been associated with environmental factors, such as mode of delivery, breastfeeding and early exposure of antibiotics (25–29). Although our understanding of the exact mechanisms of how certain microbes provide protection is still limited, recent research suggests that antibodies play a role in shaping the microbiome and thus indirectly contribute to the development of allergies (22, 30, 31). In this review, we aim to summarize recent work on the development of the microbiota-immune axis in early life and its potential involvement in the onset of allergies in early life.

## 2 The microbiome in early life

### 2.1 Development of bacterial communities

The bacterial microbiome composition in early life has a high microbial diversity and strain heterogeneity, as well as a high turnover, as only about 11% of bacterial colonizers persist past the first year of life (32). Research suggests that strains of microorganisms contributing to the infant microbiome originate from a variety of maternal sources and include vaginal, dermal, oral and intestinal communities (9, 33, 34). Those microbial species are thereby mirroring the mode of delivery, which results in a noteworthy variance in microbial gut composition post-birth and has a distinct colonization pattern characterized by a limited number of species (6, 9, 35, 36). Nevertheless, the influence of the maternal microbiome decreases within a few days after birth (37). The microbial composition is declining dramatically within the first week after birth, followed by a recovery and gradually increases again over the following months (37). In vaginal deliveries, *Lactobacillus* is the predominant species in the infant's oral, cutaneous and intestinal environment, accompanied by *Senathia* and *Prevotella*, exhibiting a similar microbiota profile to the vaginal environment (38, 39). Conversely, the gut microbiome in neonates delivered by cesarean section (C-section) is associated with reduced microbial diversity, and mainly colonization by *Staphylococcus*, followed by *Propionibacterium* and *Corynebacterium*, resembling the microbial composition of maternal skin (6, 40). In one study, infants born vaginally shared 72% (135/187) of gut microbes with their mother, whereas those born by C-section only shared 41% (55/135) (9). Beyond maternal transfer of microbes, the newborn undergoes a gradual colonization by environmental microbes while also being introduced to potential pathogens (41). While the mode of delivery has a significant impact on the initial seeding of the gut microbiome, soon the feeding mode rapidly becomes more influential in shaping its composition. Breastfeeding promotes colonizing of beneficial microorganisms, but does not seem to fully compensate the deficiency of *Bifidobacteria* in infants delivered through C-section (42, 43). However, generalizing infants’ microbial compositions remains challenging due to the high variability across different cohorts, considering the differences in geographic and cultural settings (Table 1).

**Table 1:** Metagenomic studies investigating microbiome development in early life.

	Publication	*n*	Timepoints	Duration of study	Mother/child	Method	Info
Bäckhed et al. (9)	Dynamics and stabilization of the human gut microbiome during the first year of life, cell host & microbe (2015)	98 full-term infants + mothers	Mother: 2 days after birth; Child: birth-4 months–12 months	12 months	Yes/Yes	Shotgun metagenomic sequencing	How the gut microbiota develops during the first year of life after a normal term pregnancy
Barker-Tejeda et al.	Comparative characterization of the infant gut microbiome and their maternal lineage by a multi-omics approach, nature communications (2024)	16S S rRNA: Infants (0–12 months old) *n* = 69, mothers *n* = 67, grandmothers *n* = 64 Shotgun metagenomics: 40 Infants, 45 Mothers, 43 Grandmothers (*n* = 128)	Once	Once	Yes/Yes	16S rRNA/shotgun metagenomics	Characterization of the fecal microbiome and metabolome of infants, their mothers, and grandmothers
Bergström et al. (35)	Establishment of intestinal microbiota during early life: a longitudinal, explorative study of a large cohort of Danish infants, applied and environmental microbiology (2014)	300 full-term infants	9, 18, and 36 months	3 years	No/Yes	16S rRNA	The formation of gut microbiota during the first 3 years of life. Goal was to identify correlations with dietary habits and physiological parameters. Focus on the development of body weight
Davis-Richardson et al.	Bacteroides dorei dominates gut microbiome prior to autoimmunity in Finnish children at high risk for type 1 diabetes, frontiers of microbiology (2014)	76 full-term infants	4–6 months until 2.2 years of age, in monthly intervals	2 years	No/Yes	16S rRNA	Early changes in the microbiome may be useful for predicting type 1 diabetes in genetically susceptible infants
Garmaeva et al. (33)	Cransmission and dynamics of mother-infant gut viruses during pregnancy and early life, nature communications (2024)	32 infants/30 mothers	Gestational weeks 12 and 28, at birth, and months 1, 2, 3, 6, 9 and 12 after birth	12 M	Yes/Yes	Shotgun metagenomic sequencing	Infant gut virome is dynamic in the first year of life and is influenced by feeding mode and place of delivery
Hoskinson et al.	Delayed gut microbiota maturation in the first year of life is a hallmark of pediatric allergic disease, Nature communications (2023)	1,115 infants	After 3 months/1 year	5 years	No/Yes	Shotgun metagenomic sequencing	The maturation of microbiota is associated with the infant gut metabolome and subsequent allergy development
Liang et al. (55)	Step-wise assembly of the neonatal virome modulated by breastfeeding, Nature (2020)	20 mother infant pairs	After birth (0–4 days), 1 and 4 months	4 months	Yes/Yes	16S rRNA	Assembly of the viral community in neonates takes place in distinct steps
Lou et al. (32)	Infant gut strain persistence is associated with maternal origin, phylogeny, and traits including surface adhesion and iron acquisition, Cell reports medicine (2021)	42 infants + 29 mothers (23 full-term and 19 preterm infants)	0–4, 8, and 12 months	1 year	Yes/Yes	Shotgun metagenomic sequencing	Approximately 11% of early microbial colonizers*,* persist during the first year of life
Roswall et al.	Developmental trajectory of the healthy human gut microbiota during the first 5 years of life, cell host microbe (2021)	471 infants	4 and 12 months and at 3 and 5 years of age	5 years	Yes/Yes	16S rRNA	Gut microbiota mature along similar trajectories but at different speeds and gut microbiota has not yet reached adult complexity in 5 years old children
Stewart et al. (36)	Temporal development of the gut microbiome in early childhood from the TEDDY study, nature (2018)	903 infants	Months 3 to 46 of age	3.8 years	No/Yes	16S rRNA/shotgun metagenomics	Developing gut microbiome undergoes three distinct phases of microbiome progression
Stokholm et al. (25)	Maturation of the gut microbiome and risk of asthma in childhood, Nature communication (2018)	690 infants	1 week, 1, 3, 6, 12, 18, 24, 30, and 36 months, and yearly thereafter	<5 years	No/Yes	16S rRNA	One year old children from asthmatic mothers with altered microbiome had increased risk of asthma at 5 years of age.
Wernroth et al. (46)	Development of gut microbiota during the first 2 years of life, nature scientific reports (2022)	83 mother infant pairs	Birth, 6, 12, 24 months; gestational week 26–28, and 6 months post-partum	2 years	Yes/Yes	16S rRNA	Gut microbiota in infants is low in diversity with differences across individuals with regards to composition. Perinatal factors attenuate with age
Yatsunenko et al. (11)	Human gut microbiome viewed across age and geography, nature (2012)	326 individuals aged 0–17 years (83 Malawian, 65 Amerindian and 178 residents of the USA)	Once	Once	Yes/Yes	16S rRNA	Differences in bacterial compositions and functional gene repertoires between infants from different countries
Zeng et al.	A compendium of 32,277 metagenome assembled genomes and over 80 million genes from the early-life human gut microbiome, nature communications (2022)	6,122 infants	>3 years of life	Once	No/Yes	Shotgun metagenomic sequencing	Early-Life Gut Genomes (ELGG) catalog with 32,277 genomes representing 2,172 species from 6,122 fecal metagenomes

#### 2.1.1 Longitudinal development of the gut microbiota and links to diseases

**Figure 2:**
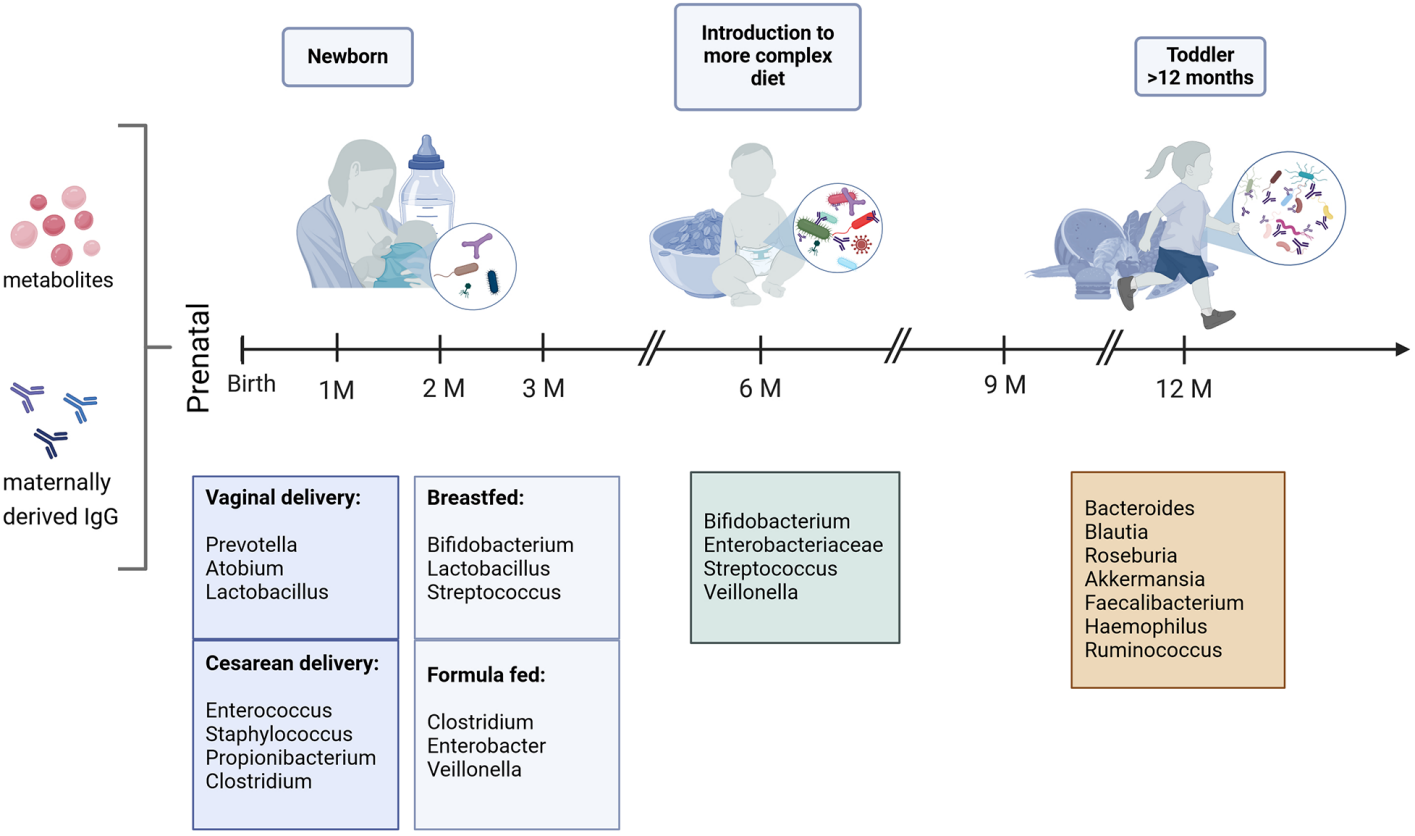
Predominant bacterial genera in the first year of life. Bacterial composition is subject to constant fluctuations, mostly influenced by delivery- and feeding mode in the first months of life. It gradually approaches the adult state starting with the introduction to solid food (9, 36, 46). Created with BioRender.com.

A longitudinal study by Stewart et al. (36) analyzing the bacterial gut microbiome of over 900 children from four high income countries (Germany, Finland, Sweden and the United States) from age three months to 46 months has shown, that the developing gut microbiome undergoes three different waves of development. The first wave is called developmental phase (month 3–14), followed by a transitional phase (month 15–30) and a stable phase (months 31–46). The birth mode has a significant association with the gut microbiota composition in the developmental phase, clearly indicated by the higher levels of *Bacteroides spp.* in infants born vaginally (36). Overall, the first gut colonizers are aerobic and facultative anaerobic bacteria (*Enterobacter, Enterococcus* and *Escherichia*) as well as Firmicutes (*Streptococcus* and *Staphylococcus*). As mentioned before, breastfeeding is the most significant factor influencing the gut microbiota composition and is associated with high levels of obligate anaerobe Actinobacteria such as *Bifidobacterium* species. In the developmental phase of the gut microbiome, the infants’ gut is colonized with primary *Bifidobacteria* which are responsible for human milk oligosaccharides (HMOs) catabolism and have genes involved in plant polysaccharide metabolism (44). These high levels of *Bifidobacteria* in this phase of development protect against allergy (45). Overall, an increased alpha diversity (describing the diversity of species within an individual), and a reduced beta diversity (defined as the diversity between different individuals), can be observed in the growing infant, with the microbiota becoming more complex over time (9, 11, 46, 47). Cessation of breastfeeding can be linked to a faster maturation of the gut microbiome, marked by the phyla of Firmicutes (*Lachnospiraceae* and *Ruminococcaceae*) and Bacteroidetes (*Bacteroidaceae*) (11, 36, 44). The introduction to solid food is marked by an increase in alpha diversity, mainly Actinobacteria, Bacteroides, Firmicutes, Proteobacteria and Verrucomicrobiota (36). The transitional phase (months 15–30) is characterized by a decrease of *Proteobacteria* and a strong increase in *Bacteroidetes* that proceeds to the stable phase (months 31–46), which, as the name would suggest, is characterized by unchanged alpha diversity and composition (36). These observations are in line with other metagenomic studies (9, 32, 46). Overall, the infant microbiome is subject to constant fluctuations and gradually approaches the adult microbiome at around 2–3 years of age (Figure 2) (48).

The composition of the bacterial gut microbiome in early life is linked to various diseases and, presumably, the starting point of immunological imprinting of allergies, highlighted by various studies with *Bifidobacterium spp* (22, 30). Isolation of *Bifidobacterium breve* and *Bifidobacterium longum subsp. infantis* from human feces stimulated T-regulatory (Treg) cell accumulation and thereby protected against allergy in mice. Moreover, *B. longum subsp. infantis* supplementation resulted in elevated levels of indole-3-lactic acid (ILA), which has the ability to dampen T-helper 17 (Th17) and T-helper 2 (Th2) cell responses (49). Furthermore, *B. longum subsp. infantis* not only has the ability to increase the number of Tregs, but has also been shown to have protective effects against asthma by mitigating the effect antibiotics in early life (45, 49). Furthermore, within the first year of life, *Bifidobacterium breve* colonization is linked to a reduced risk of atopic dermatitis, whereas *Bifidobacterium catenulatum* is associated with an increase (50). Interestingly, several studies observed that having a furry pet living in the household was associated with a lower relative abundance of the genus *Bifidobacterium* (36, 46, 51). Moreover, an immature microbial composition in the gut has been linked to an increased risk of asthma at age five, with lower abundances of the genera *Firmicutes* families like *Lachnospiraceae* and *Ruminococcaceae* (25, 52).

### 2.2 The virome in early life

In addition to bacteria, the human gut contains an enormous number of viruses, including eukaryotic viruses as well as bacteriophages (viruses infecting bacteria) (18, 19, 33). The complex interplay between the vast array of intestinal viruses (termed the “virome”) and the immunological maturation of children during their first years of life is an increasing area of research (18, 33, 53–57). Despite the overwhelming prevalence of viral entities, especially when compared to the bacteriome, our understanding of their role is limited. The maternal virome is stable in its composition, at least in the time from late pregnancy and after birth (33, 53). As more and more research focuses on influences of the intestinal virome in early life development, it has been observed that virus composition underlies strong fluctuations depending on various environmental factors such as age at gestation, older siblings, type of birth, feeding pattern, and geographical location or being born during summer (33, 58–61).

As soon as the infant is born, the varying impact of different factors becomes apparent. At birth the delivery mode has the highest impact on the predominance of the gut virome (infants born vaginally showed higher diversity compared to infants born by C-section). After one and three months after birth, the largest effect was the gestational age at birth (preterm vs. term) (57). Overall, this first phase of viral colonization is characterized by the induction of prophages from pioneering bacteria followed by the colonization of viruses infecting human cells, which is regulated by breastfeeding (55). Breastfed infants showed lower numbers of viral operational taxonomic units (vOTUs) based on bulk metagenomes, compared to partially or exclusively formula-fed infants. Formula-fed infants also show a higher alpha diversity in their virome (33, 57). The feeding mode had a comparable effect size to the delivery method at month three but overtakes in the following months in breastfed infants. The geographical region becomes relevant from month six onward, with influences manifesting through the introduction of solid food to the infants’ diet (57). While the transmission of eukaryotic viruses such as cytomegalovirus, herpes simplex and rubella virus is known, transmission of bacteriophages from mothers to their offspring has still rarely been systematically addressed (62).

Bacteriophages constitute the main source of viruses in the gut (63). They demonstrate remarkable diversity within the gut milieu, mirroring the prevailing composition of gut bacteria and being key players of the modulation of the bacterial gut microbiome (56). Following birth and extending through the first two years of life, bacteriophage composition undergoes significant changes and a rapid expansion linked to the increase of bacterial communities in the infants gut (54, 57). However, this expansion is followed by a notable reduction shortly thereafter, resembling an adult like state by two to three years of age, accompanied by a decrease in diversity being inversely correlated with the bacterial diversity (33, 64, 65). While the maternal virome predominantly comprises bacteriophages and exogenous viruses from the environment and diet, the neonatal counterpart is composed of a unique set of bacteriophages (53). Intriguingly, depending on the study cohort, only 15%–32.3% of this virome diversity is likely to be maternally derived, for instance during vaginal delivery and breastfeeding (7, 33, 58, 66). Some distinct bacteriophages, for example, those that infect *Bifidobacteria*, can be transmitted from mother to child (67). Nevertheless, environmental transmission seems to be the major route in transmission (53). They most likely originate from maternal skin, infectious exposures as well as contaminated surfaces (53, 58, 68). Overall, each infant's virome exhibits a distinct signature, although monozygotic twins manifest a higher interspecies similarity compared to unrelated infants (58, 65, 69).

Recent research showed, that the infant gut virome is dominated by a rich temperate phage community, which is able to integrate their genomes into the chromosomes of their bacterial host. Deficiencies in certain temperate phage families could increase the risk of asthma development by one year of age, independent of their bacterial hosts (70, 71). The constant changes in the composition of the gut virome during early life significantly shape the gut bacterial microbiome, a factor known to influence the development of allergies. Despite this potential link, our understanding of the specific role of the gut virome in the development of allergies is still limited. However, there are correlative hints. Leal Rodríguez *et al*. have reported, that an exposure to cats, not dogs, in early life was associated with an asthma virome fingerprint, along with a negative correlation with being born in summer and having older siblings (61).

## 3 Pre- and postnatal factors in early life immune development

### 3.1 Prenatal factors of immune development

During pregnancy, rejection of the semi-allogeneic fetus is avoided by an immune privileged status of the placental trophoblast, a vascular separation from the mother and various maternal tolerance mechanisms (72, 73). Thus, the success of human pregnancy depends on maintaining a subtle balance between two conflicting aspects of the immune system. The fetus must acquire the ability to accept both its own and maternal antigens, while building protective immunity in anticipation of birth (74, 75).

Therefore, the placenta plays an essential role in the development of the fetus. The existence of a placental microbiome still remains a debated topic within the scientific community and it is currently rather believed that the infant encounters the first microorganisms during delivery (76–78). In the womb, the fetus is assumed to live in a largely sterile environment and is protected from infection by the maternal immune system (79). Chorionic villi, forming at the end of the first trimester of pregnancy, create a maternal-fetal interface. These villous structures, resulting in a hemochorial placenta, allow maternal blood to directly come into contact with the fetus via its fetal derived placenta. These structures not only provide the developing child with nutrients and oxygen, but they also facilitate the transfer of antibodies from maternal blood flow across the syncytiotrophoblast layer of the chorion into the inner layer of cytotrophoblast precursor cells (80, 81). From there, antibodies can travel via the FcRn into the fetal capillaries providing a layer of defense against pathogens (82).

In addition, microbial antigens and metabolites are transferred across the placenta, and thereby able to prime the fetal immune system (83, 84). Transferred metabolites are currently getting attention for their role in atopy protection (85). They have been demonstrated to affect transcription of the target gene Foxp3 in the lung, which has been associated with asthma development (86). The fetal innate immune system is being prepared for the subsequent influx of microbes that will later colonize the infants’ intestine and thereby influence the leukocyte development (87–89).

### 3.2 Adaptive immune response in early life—how the mother prepares the unborn child for a life with microbes

During the final trimester of pregnancy, a significant increase in maternally transferred IgG levels can be observed (90). Maternal antibodies, spanning all subclasses of IgG, are then transported across the placenta to the developing fetus. This transfer is facilitated by the FcRn ensuring the efficient passage of antibodies from maternal circulation to the fetus (91–93). Although the FcRn binds to the CH3 domain of the Fc fragment of IgG antibodies, previous studies reported a hierarchical transfer of different IgG subclasses independent of their specificity in with IgG1 is preferentially transferred across the placenta (IgG1 > IgG4 > IgG3 > IgG2) (94, 95).

More recent studies suggest a preferential transfer depending on Fc-glycosylations to the fetus, and thereby the transfer of more functionally enhanced antibodies (96–99). Thus, glycosylations could mediate the binding of IgG to certain placental Fc receptors, including FcγRI, FcγRII, and FcγRIII, allowing a more efficient transfer (99, 100). One indication of this can be seen in premature infants, which, despite having an overall limited number of total maternal IgG, possess similar anti-viral and neutralizing antibodies mirroring a robust transfer of broadly reactive and functional relevant antibodies (101). This finding suggests that the transfer of the most functional antibodies occurs very early in pregnancy, although the exact mechanisms of selection are not yet known. However, it appears, that the placenta preferentially transfers antibodies eliciting innate immune effector functions activating natural killer cells in the earliest days of life after birth (98).

Recently, Dolatshahi et al. (82) conducted a longitudinal study investigating the humoral immune response against vaccine or pathogen derived antigens in 12 full-term (FT, gestational age 37–40 weeks) and 11 preterm infants (PT, gestational age 24–29 weeks). Among mothers of both groups, they observed significant heterogeneity in IgG, IgM, and IgA levels, as well as Fc receptor binding antibodies. IgG subclasses were detectable in cord blood but declined in the following weeks, consistent with expectations. Comparing mother-baby dyads, differences in transfer of antigen-specific antibodies from the mothers to the children across different gestational ages could be observed. Cord blood of FT babies showed an enrichment of certain antibody populations, with enhanced levels of total IgG against norovirus, the tetanus toxin, *Streptococcus pneumoniae*, poliovirus, hepatitis A, the mumps toxin and the allergens Ara h 2 (peanut allergen) and Bet v 1 (birch pollen allergen). The predominant allergen-specific antibodies in preterm babies, were specific against Bos d 8 (bovine milk allergen). Notably, one week after birth, the only significantly enriched antibodies were specific for cow milk in PT compared to FT, setting a potential link for antibodies as a surrogate of food allergy (82). Furthermore, these findings suggest the presence of antigen-specific differences in transfer rates across the umbilical cord, possibly selected not only by the Fc but in collaboration with the Fab domain (82, 102, 103). Although the antibody profile across infants began to normalize after three months, the antibody profiles of FT and PT infants still differed. Few but potentially important antibody specificities were observed in FT vs. PT infants. These were the persistence of *S. pneumoniae*-and peanut-specific antibodies, as well as antibodies against adenovirus, cytomegalovirus and polio. This observation may suggest an explanation for the enhanced susceptibility to infections in PT infants. Maternally derived antibodies decreased to very low levels by three months of age, with a more pronounced decline in PT infants. However, the decline in transferred functional antibodies was similar in FT and PT infants. The FcRn-binding antibodies against the bovine milk allergen increased slightly in both infant groups by that time, along with an increase in IgM and IgA1 concentrations (82). It can thereby be concluded that elevated risks of viral infections in premature infants cannot be attributed to the lack of maternal antibodies, but rather to the weaker mucosal barriers and contact to disease-associated environmental exposures, e.g., due to longer stays in the hospitals, especially in intensive care units (101). Despite an adjustment of the adaptive immune response comparing PT and FT, very PT (<31 weeks) seem to have a more elevated risk in asthma (approximately 3.6 times higher than FT) (104). Overall, any observed differences in the composition of the microbiome, disappear after six months when the infant is getting introduced to a more complex diet (9).

### 3.3 Lactation

#### 3.3.1 Breastmilk

Breastmilk is a vital factor in shaping the composition of the gut microbiota and thereby influencing the development of the immune system in infants (36, 105). Milk produced during the first days after infant birth is called colostrum and is characterized by its notably higher protein content and reduced levels of carbohydrates and fat compared to more mature milk, indicating a primary immunological role rather than a nutritional one. After four to five days, the content ratios start to change, by lower concentrations of immunoglobulins and proteins, however, the overall composition remains similar. Human milk is characterized by the presence of essential components altering cellular differentiation and gene expression such as lactoferrin, and short-chain fatty acids, thereby directly influencing immune cells to a more tolerogenic-like response via transforming growth factor β (TGF-ß) and interleukin 10 (IL-10) (106, 107). Moreover, breastmilk contains leukocytes and vital immunoglobulins, including IgA and IgM, as well as IgG. Soluble IgA (sIgA) stands as the predominant immunoglobulin in human milk, constituting over 90% of the total amount. It is followed by IgM and IgG, whose concentrations increase in more mature milk (108–111). As reviewed by Rio-Aige et al. (111), concentrations of sIgA are in a wide range from around 7.5 g/L in colostrum and 1.6–2 g/L in more mature milk. It is generated by plasma cells that migrate from the mesenteric lymph nodes to the mammary glands during the later stages of pregnancy and throughout the lactation period. Common in all mucosal secretions, it has the ability to neutralize pathogens before they come in contact with epithelial cells, thus preventing inflammation and damage to tissues (112). IgA antibodies have been shown to anchor beneficial bacteria in the mucus layer of the intestine, thereby promoting the colonization of the child's gut with a diverse set of microbiota (113).

Nonetheless, human breastmilk is highly personalized as its composition varies highly between mothers. The microbial composition of breastmilk is reflected by differences dependent on the mode of breast milk feeding, nursing directly from the breast vs. using pumps or by bottle-feeding (114). The precise origins of milk microbiota are currently subject to debate, although evidence suggests that the entero-mammary pathway or retrograde translocation may serve as significant routes for microbial colonization (115).

The HMOs found in breast milk not only serve as an optimal source for bacteria, but also have other important functions. Some HMOs provide support in maintaining structure and function of mucosal gut tissues, as well as, together with sIgA, preventing necrotizing enterocolitis (NEC), an acute inflammatory bowel necrosis, affecting the colon in neonates, especially in PT children (116, 117). *Bifidobacteria*, the most extensively studied among HMO-fermenters, are closely linked to breastfeeding. These microorganisms have the unique capability to convert aromatic amino acids like tryptophan, tyrosine, and phenylalanine into their respective lactic acid derivatives using aromatic lactate dehydrogenase (ALDH). Indoleacetic acid derived from tryptophan has been demonstrated to activate the aryl hydrocarbon receptor (AhR), which plays a pivotal role in regulating intestinal homeostasis and immune responses. In addition, it drives the upregulation of immunoregulatory molecules in CD4^+^ *T*-cells, which reduces the differentiation of Th2 and Th17 cells (45, 118). Moreover, depletion of *Bifidobacteria* was shown to be a marker of systemic and intestinal inflammation and increased the risk of developing autoimmune diseases and atopic wheeze (45, 119, 120).

#### 3.3.2 The introduction of solid food and the weaning reaction

The first major immune response to colonizing microbiota after birth begins at the time of weaning and the introduction of solid food (121). It is resulting in a notable augmentation in both the quantity and diversity of bacterial taxa in the gastrointestinal tract. Notably, murine studies have demonstrated that throughout the weaning period, when levels of the epidermal growth factor (EGF) in breastmilk start to decline, goblet cells exhibit an enhanced capacity for the translocation of antigens from the intestinal lumen into the lamina propria (122). In response to this influx of luminal antigens, the neonatal immune system orchestrates a robust production of cytokines and T-cells lean towards an immunoregulatory phenotype, thereby helping to establish long-term tolerance to commensal microorganisms (121, 122). Increased food diversity is negatively correlated with the development of asthma and food allergy up to year six. Furthermore, increased isotype switching to IgE and a reduced expression of Foxp3, which is associated with Treg expression and moreover associated with a low food diversity score (123). In addition, certain dietary vitamins appear to be of great importance in the prevention of pathological imprinting in early life. Dietary vitamin A-derived retinoic acid holds a protective effect by inducing ROR*γ*t^+^ Tregs during the weaning response, as well as riboflavin metabolites during the neonatal period by generating mucosal-associated invariant T (MAIT) cells (121, 124). This immunological response can be modulated through temporary antibiotic intervention or excessive caloric intake, as has been shown in mice. These disturbances lead to a higher susceptibility to inflammatory pathology characterized by high release of cytokines (121, 125).

## 4 Postnatal immunity

In the waves of fetal hematopoiesis, from the yolk sac (126, 127) (in humans at four weeks, in mice at embryonic day seven to nine) to the fetal liver (127, 128) (in humans at six weeks, in mice at embryonic day twelve) and to the bone marrow (128) (in humans at 10 weeks, in mice at embryonic day 7–15), specific immune cells develop that are important for early life tolerance against microbiota. Neonatal B and *T*-cells show more innate-like functions, as they have the ability to respond to antigens more quickly than the adult version. Unconventional B- and *T*- cell subsets have been shown to be particularly responsive by early life microbial exposure and metabolites, whereas the exposure to pathogens in early life can have a strong influences on the development and the functionality of the immune system (129).

### 4.1 Immune interactions by gut microbiota, infectious diseases and metabolic effects

Once the infant is getting colonized with microbiota, the adaptive and innate immune system must adjust to tolerate this intricate interaction. In order to recognize bacteria, the innate immune system uses various receptors, including pattern recognition receptors (PRR) like Toll like receptors (TLRs) and nucleotide-binding oligomerization domain/caspase recruitment domain (NOD/CARD) isoforms. These receptors are expressed by surface enterocytes and dendritic cells and are crucial for bacteria- host communication (130). Thus, PRRs can detect conserved microbe-associated molecular patterns (MAMPs), such as components of the bacterial cell wall, like lipopolysaccharides (LPS), peptidoglycan and flagellin. By binding TLRs, the microbiota can suppress inflammatory responses and promote immunological tolerance (131, 132). This sensor function of TLR's as well as downstream signaling molecules are fully developed in newborns (133). Following initial exposure to LPS shortly after birth, intestinal epithelial cells exhibit a reduced response to subsequent TLR stimulation, thereby favoring microbial colonization and maintaining host-microbe homeostasis (134). Following stimulation by TLRs, a spectrum of cytokines is synthesized that regulate both the adaptive and innate immune systems during ontogeny (135). In PT infants, the predominant cytokine profile is biased toward the production of anti-inflammatory mediators, particularly IL-10. In contrast, the predominant cytokine in term infants tends to promote T-helper 17 (Th17) cells, characterized by elevated levels of interleukin-6 (IL-6) and interleukin-23 (IL-23) (136–138). IL-12p70 is one of the final cytokines to reach adult levels following TLR stimulation as it is promoting the development of Th1 cell immune responses (138).

Regarding adaptive immunity, a reduced diversity in the gut microbiota has been linked to the development of allergy (139, 140). This reduction can be provoked by treatments with antibiotics, but the exact mechanisms are not yet known (15, 141). One possibility could be that microbes induce the T-helper 1 (Th1) pathway as well as Tregs, thereby counteracting the Th2 cell responses associated with allergy (142, 143). For example, endotoxin, produced by gut bacteria in early life is linked with Th1 maturation and prevents from Th2-mediated responses in a mouse model of asthma (31).

Exposure to pathogens in early life can have a great influence on the development and the functionality of the immune system. Certain infections can lead to life-long pathologies affecting all organs and influence the onset of various diseases (144). For instance, early life infection with *Listeria monocytogen*es can cause long term-organ specific alterations in both the innate and adaptive immune system (145). Similarly, some infections by enteroviruses contribute to the onset of autoimmune diseases, such as diabetes (146–148), while others, like Respiratory syncytial virus (RSV) (149, 150), *Streptococcus pneumoniae* (151) and Rhinovirus (152, 153) show a strong association with increased allergic airway diseases in adulthood.

In addition to the immune system, also metabolites play an important role in early life microbiota-host interaction. Alongside to the aforementioned HMOs (see previous section “Breastmilk”), SCFAs (short chain fatty acids) produced by gut microbiota (including acetate, propionate and butyrate), are key metabolites linked to gut colonization and immune maturation. SCFAs are produced by anaerobic bacteria that ferment complex carbohydrates originating from diet and colonic mucus (154). Reduced levels of SCFA-producing bacteria (such as *Ruminococcus bromii* and *Faecalibacterium prausnitzii*) have been shown to be associated with an increased risk of allergic diseases in infants, as they promote anti-inflammatory and tolerogenic immune responses (20, 155). Additionally, they are involved in Treg differentiation, as demonstrated in both murine (156–158) and human cells (159).

### 4.2 Environmental influences and the hygiene hypothesis

Several epidemiological studies have demonstrated a link between growing up in specific agricultural environments and being protected from allergies in childhood. This protective effect has been attributed to respiratory exposure to certain environmental microbes (160–163). The hygiene hypothesis, first postulated by Strachan (164) more than 30 years ago, gives an explanation for this connection. According to this hypothesis, a decreased frequency of infections directly contributes to the increase in allergic and autoimmune diseases (165). Repeated low grade acute immune responses triggered by infectious and even harmless microbes in early life are associated with lower prevalence of chronic inflammatory disorders in adulthood (160, 166). Prenatal exposure to household pets, especially dogs, has been shown to lower the risk for asthma and atopic dermatitis until 2 years of age (167). As it was shown by Panzer *et al*. (168), prenatal, as well as early life dog exposure is associated with an altered gut microbiome during infancy, supporting a potential link between dog-keeping and a decreased allergy risk.

Several farm derived bacteria have been reported to contribute to the protection against asthma, including *Lactococcus lactis* (169), *Staphylococcus sciuri* (170), *Bacillus licheniformis* (171) *or Acinetobacter lwoffii* (172–174)*. Acinetobacter lwoffii* has been suggested to induce pro-inflammatory responses in airways by increasing IL-6 (172). Epigenetic modifications in CD4+ *T* cells then result in IL-10 induction. The combined activity of IL-6 and IL-10 influences the gastrointestinal microbiome, with specific taxa being significantly associated with either disease activity or protection (172).

Furthermore, certain biochemical modifications of the genetic information carrying chromatin, called epigenetic modifications, have been associated with the increase of allergic diseases. They are not changing the nucleotide sequence of the genome, but are best known for changing the accessibility of genes, thereby regulate the gene expression (14, 175). Those modifications can be induced by several extrinsic factors interacting with the genetic background (14). In particular, dietary components in breast milk and bovine milk, exposure to microbial components, house dust, as well as the production of SCFA by gut microbiota are associated with several epigenetic modifications in the gene expression (153, 176). Moreover, maternal exposures during pregnancy have been shown to influence the immune development *in utero* by epigenetic mechanisms and thereby affecting the onset of allergy (177–179). The differentiation of Th cell populations is strictly controlled by epigenetic mechanisms, which control the differentiation into the with allergy associated Th cell populations (14, 180). Allergy is specifically associated with changes in DNA methylation patterns in the Th2, Th1, Th17 and Treg subsets (180).

In the next chapters, we highlight lymphoid immune cells which contribute to an establishment of mucosal immune-microbiota homeostasis such as B-1 cells, RORγt^+^ Tregs, MAIT cells and invariant natural killer T (iNKT) cells (181).

### 4.3 B-cells in early life

Mature fetal B-cells develop in the fetal liver from post conception week nine and later in the bone marrow (127, 182). These B-cells achieve their repertoire in early stages, which is defined by somatic recombination of immunoglobulin genes and Ig heavy chain class-switching, although the formation of germinal centers and the accompanied somatic hypermutation starts by antigen exposure after birth (183–185). In early gestation, innate-like B-1 cells prevail, being the most abundant B-cell population in the peritoneal and pleural cavities. B-1 cells are thought to recognize surface epitopes of common pathogens and self-antigens and most importantly, are thought to represent the only B-cells in adult repertoires shaped by early life antigen exposures (186–189). Furthermore, B-1 cells have an pre-activated phenotype that is also conserved under germ free conditions, making them prepared for antibody secretion (190). B-1 cells can spontaneously differentiate into plasma cells and are believed to be the major source of secreted IgM in unchallenged mice (189). New *et al*. (186) showed, that neonatal immunization with group A Streptococcus recruits unique B-1 memory cells, which cannot be found when mice were immunized in adulthood. This result was confirmed by Vergani *et al*. (190), who showed that mice orally infected with a murine rotavirus as five-day-old-neonates- in contrast to those infected as adults- exclusively generated IgA plasma cells originating from early life origin (ELO)-B-cells nine weeks after infection. These plasma cells arose from the same hematopoietic progenitor cells as B-1a cells, suggesting that neonatal exposure to antigens uniquely primes immune responses later in life and ELO-B-cells harbor the memory of neonatal antigen exposure in the gut (190).

#### 4.3.1 Mucosal IgA serves as the first line of defense against invaders

IgA serves as the first immune protection against invading pathogens and represents the predominant antibody isotype in mammals at mucosal surfaces, while IgG dominates systemically in blood. It is secreted across mucosal surfaces and the intestinal epithelium, especially in the small intestines. Polymeric IgA is transcytosed through epithelial cells from the basolateral surface by the polymeric immunoglobulin receptor (pIgR) (191). As early as 1996, it has been shown that a significant portion of bacteria found in feces is bound by IgA, showing a continuous presence of IgA antibodies in response to the persistent resident microbial population (192). This type of antibody coats and agglutinates microbiota and antigens coming from components of the lumen as well as toxins of the intestine to prevent direct interaction with the host (193, 194). Moreover, it has the ability to preferentially coat colitogenic bacteria, and thereby prevents inflammation and maintain intestinal health. Impairment of IgA secretion is associated with increased susceptibility to various diseases of the gut, *e.g.,* enterocolitis (195). IgA assumes a crucial role in the regulation of bacterial gene transcription, ultimately influencing the composition, invasiveness, and immunometabolic functions of bacterial communities within the gut (196–198). Conversely, commensal bacteria have the capacity to stimulate the production of IgA antibodies (199). These IgA antibodies play a crucial role in boosting the humoral mucosal immune defense system. Notably, they are significantly decreased in germ-free (GF) animal models, which can be reversed by initiation of microbial colonization (200).

IgA can be produced via two distinct pathways. The first, the *T*-cell-dependent pathway, yields high-affinity antibodies that are predominantly directed against specific protein antigens, particularly from pathogens. These responses occur in germinal centers in gut-associated lymphoid tissues, *e.g.,* Peyer's patches and mesenteric lymph nodes. The second, termed as the *T*-cell-independent pathway, operates primarily through specialized B-cells in the small intestine. These B-cells in both, organized lymphoid tissues and non-lymphoid tissues, are utilizing innate immune receptors, for example TLRs (201–203). The *T*-cell-independent pathway generates lower-affinity antibodies that recognize a wider variety of microbial antigens. IgAs yielded from both pathways are directed against commensals, but to different strains and species (204).

Infants initiate the production the of IgA between two and four weeks of age (205). However, it appears that the *T*-cell independent pathway holds greater significance in children until they develop an adult-like microbiome and establish germinal center-dependent IgA production (206). Moreover, experiments in mice have demonstrated that the production of neonatal IgA in pre-weaning immunocompetent pups is notably increased when they are fed with milk of immunodeficient mothers. Furthermore, enrichment of special maternal derived microbiota will induce early enhanced IgA production in the intestines, as it has been specifically observed with *Limosilactobacillus reuteri* (207)*. L.reuteri* is known for its antimicrobial activity as it produces a variety of substances against gram-positive and gram-negative bacteria, fungi and parasites. This finding demonstrates that certain bacteria have the ability to influence immune responses against potentially hazardous microbiota in infants by both, direct and indirect ways (208). Beyond that, IgA has the ability to interact and thereby neutralize harmless food antigens by preventing their penetration of the gut epithelium (209). As such, it plays an important role in creating tolerance, thereby preventing allergic sensitization (210).

### 4.4 *T*-cells

Within the intestinal tract, *T*-cells play a key role in balancing the immune responses to commensal microbes by inhibiting inflammatory responses targeted against them while at the same time also preventing them to break mucosal barriers. It has been shown in mouse experiments that intestinal microorganisms are transported from the intestines to the thymus by CX3CR1+ dendritic cells, which present microbially-derived antigens to *T*-cells and thereby initiate their expansion (211). As fetal *T*-cells are hyperresponsive to foreign antigens, it is believed that those components are priming fetal memory *T*-cell differentiation (212, 213). Compared to adult *T*-cells, neonatal ones seem to be more sensitive upon antigen exposure, having distinct gene expression profiles and, being capable of shifting quickly from a pro-tolerant state to self- and non-self-antigens, while being able to mount rapid effector function in case of injury or infection (214, 215).

#### 4.4.1 Regulatory *T*- cells

**Figure 3:**
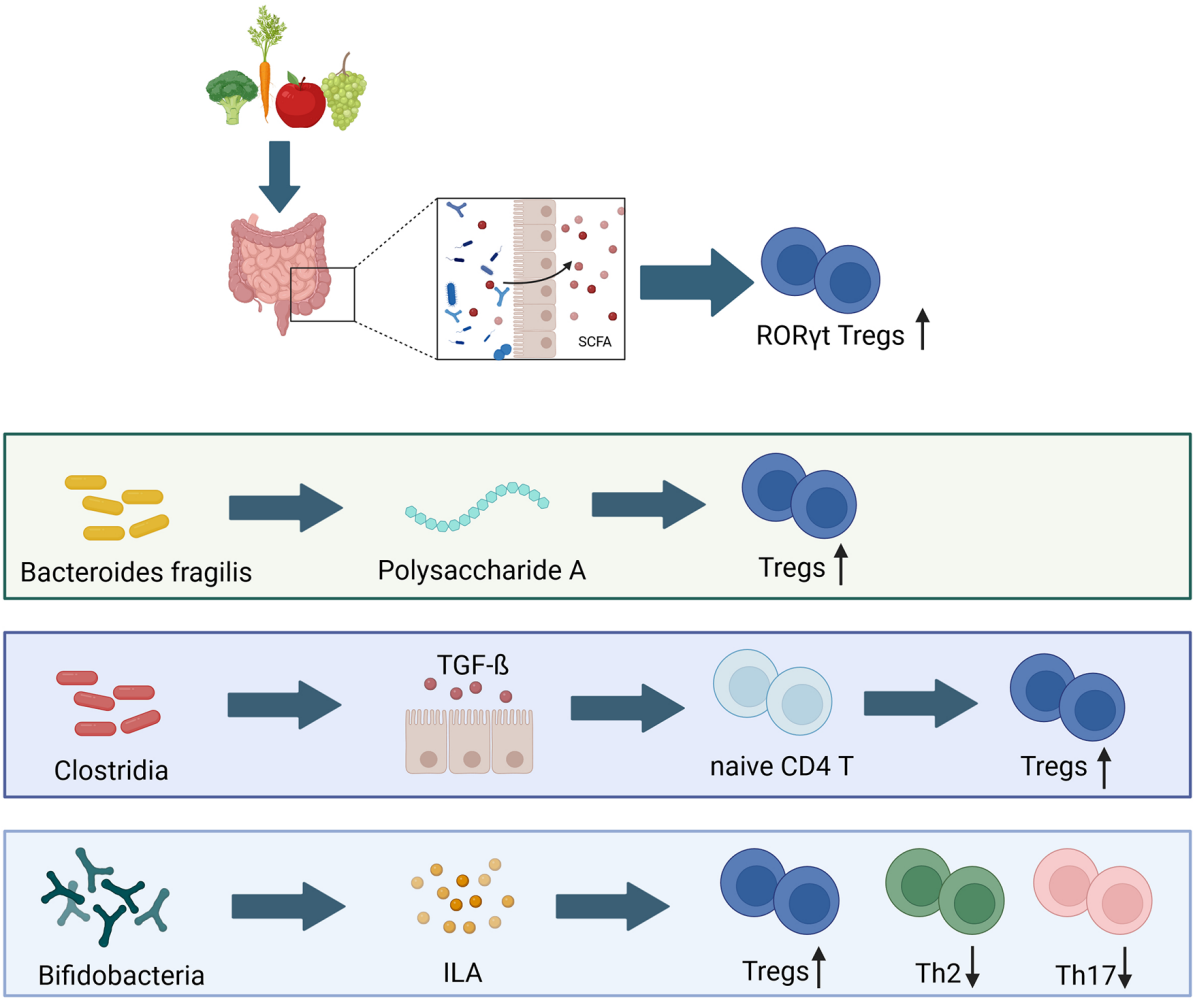
Bacterial composition has long lasting effects on early life immune development. Tregs, T regulatory cells; Th2, T-helper 2 cells; Th 17, T-helper 17 cells; SCFA, shorth chain fatty acids; TGF-ß, transforming growth factor β; ILA, indole-3-lactic acid (49, 122, 132, 221, 222). Created with BioRender.com.

The timing of antigen exposure is important. In general, fetal and neonate *T*-cells tend towards a more tolerogenic phenotype with more innate-like cytokine production than proinflammatory responses (212). Fetus-derived CD4^+^ *T*-cells preferentially produce Th2 cytokines when stimulated with low amounts of antigen (216, 217). Antigenic encounters in the phase before weaning (the window of opportunity) have the ability to induce the differentiation of neonatal CD8^+^ *T*-cells to RORγt^+^ Tregs (218, 219). Upon re-exposure to the same antigen later in life, the infant is more likely to elicit a more tolerant immune reactivity (220). *T*-cell responses to gut commensal bacteria may be dominated by a relatively small number of microorganisms and the induction of colonic Tregs depends on various commensal bacteria with different properties, including *Bacteroides fragilis* and the *Clostridium* clusters IV and XIVa. *B. fragilis* has the ability to produce polysaccharide A, thereby inducing Treg cell development via the TLR 2 (132, 221, 222). Moreover, early life mouse models have shown that these particular clostridial species are able to induce Treg accumulation in the colon following oral inoculation by inducing the release of TGF-ß and other Treg inducing factors from intestinal epithelial cells (221, 222). Furthermore, these spore forming bacteria are able to protect from colitis and elevated systemic IgE levels in adult mice (221). In humans similar effects can be observed, as children who lack Tregs in early life develop severe inflammations of the skin and the intestines following microbial colonization (223). By the time of weaning, certain dietary components, such as the vitamin A-derived retinoic acid and SCFA are linked to gut colonization and subsequent immune maturation, as they skew Tregs towards expressing RORγt^+^ and are associated with the development of asthma in later life (Figure 3) (121).

#### 4.4.2 Mucosal-associated invariant T (MAIT) cells and invariant natural killer T (iNKT) cells

The mucosal homeostasis is additionally maintained by the innate like MAIT cells and iNKT cells, both responding to glycolipids derived from early life microbial colonization. These distinct cells bridge the randomly generated T- and B-cell receptors within the adaptive immune system with the innate germline-encoded immune receptors (224). Moreover, they acquire tissue tropism, such as lung tropism, and the ability to release cytokines as part of their developmental process in the thymus before exiting the latter and accumulating in the tissue ahead of the arrival of conventional effector cells (225, 226).

iNKT cells are a rare subset of *T*-cells, having the ability to recognize self and microbial lipid antigens presented by CD1d molecules (227). They are important in influencing the outcomes of infectious and autoimmune diseases, as well as in neoplastic disorders and have been implicated in several mouse models of allergic asthma (228–231). In the first weeks of life, they migrate from the thymus to the colon and lung. Their development and residency is controlled by embryonic macrophages during a specific early life window (232, 233). The colonization by microbiota can prevent this iNKT cell migration (230). iNKT cells were shown to be the dominant CD4^+^ *T*-cell subset in airways of both, non-allergic and allergic patients with severe asthma, while not being present in the healthy population (233). An increased number of iNKT cells have been shown to being associated with asthma, possibly due to similar functions as Th2 cells (234).

MAIT cells can recognize small microbial molecules as riboflavin (vitamin B2 precursor derivates (5-(2-oxopropylideneamino)-6-d-ribitylaminouracil (5-OP-RU)) presented by major histocompatibility complex class 1b (MHC-Ib) molecule MR1 (235). As the human body cannot synthesize vitamin B2, it serves as a marker for “non-self” and has been implicated in various diseases together with bacterial dysbiosis (236–238). MAIT cells respond to various strains of bacteria and yeasts, but not to viruses (239, 240). MHC-Ib molecules have the capacity to present antigens characterized by specific amino acid sequences or chemical motifs originating from a wide range of microbiota. This highlights the potential of MAIT cells as ideal candidates for regulating communication between the microbiota and the immune system in early life (241). MAIT cells are enriched in the mucosal tissues in the intestinal tract and in the lungs, as well as in the blood and in the periphery (242–245). Although a number of immature MAIT cells can be detected in GF mice, those were not able to fully mature and expand in the periphery. Microbial colonization (and peripheral B-cells) seem to be required for the maturation and expansion, but not for the initial selection of MAIT cells (225, 246). Increased levels of MAIT cells in one-year-old children were associated with a reduced risk of asthma by year seven and a potent Th1 immune response (247).

## 5 Concluding remarks on the interplay between the microbiota and allergy

The rising prevalence of allergies has been striking over the last few decades. Several theories have been proposed to give an explanation, one of which points to higher rates of caesarean birth, formula feeding, misuse of antibiotics and general dietary changes as contributing factors. All these factors can be linked to a dysbiosis of the gut microbiome (15, 248–250).

The first potential links between allergy and microbiota appear already during pregnancy, as maternal carriage of *Prevotella copri* is associated with a decreased risk of food allergy in infants. The association is related to maternal diet, which is high in fat and fiber, the absence of antibiotics and an increased house hold size (251). Additionally, caesarean birth increases the risk of developing asthma at the age of six if the microbiota still has a C-section signature by the age of one year. However, children whose microbiota showed a non-C-section signature by one year of age, appeared to be comparable to those who were born vaginally (252). Furthermore, in the first one to three months after birth, an absence of certain bacterial taxa can be linked to an increased risk of atopy and is associated with the absence of polyunsaturated fatty acids (253, 254).

Antibiotics are one of the most commonly prescribed drugs given to children in the Western world (255). Even short administrations can cause microbial dysbiosis in the gut, which, when administered in early life, may lead to long term immunological consequences (256, 257). For instance, Vancomycin administration in early life murine models induces increased IgE levels, reduced Tregs and an overall increased risk of developing allergic asthma (258).

Legumes, such as soy, peanuts and sesame, show a significant effect on the microbiota at 12 months of age, suggesting that potential allergenic sources may contribute to allergy protective effects (259, 260). Furthermore, children with IgE mediated food hypersensitivity have a significant reduced gut microbiota diversity and richness compared to healthy children. This dysbiosis is characterized by high abundances of the phylum Firmicutes and low abundance of Bacteroidetes within a cohort of children between the age of 18–36 months. Moreover, certain enrichments of the bacterial families *Clostridiaceae*, *Ruminococcaceae*, *Lachnospiraceae*, or *Erysipelotrichaceae* were associated with milk, egg white and peanut hypersensitivities in those children (261). Taken together, there is substantial evidence that gut microbiota in early life influence the development of the immune system and dysbiosis may be involved in the onset of allergies. Future studies, both in human cohorts as well as mouse models, may shed light on the underlying mechanisms and help to establish causation.
